# Laboratory and semi-field efficacy evaluation of permethrin–piperonyl butoxide treated blankets against pyrethroid-resistant malaria vectors

**DOI:** 10.1038/s41598-022-26804-9

**Published:** 2022-12-22

**Authors:** Salum Azizi, Johnson Matowo, Njelembo Joshua Mbewe, Natacha Protopopoff, Rashid Athumani, Wambura Matiku, Magreth Shayo, Filemoni Tenu, Mark Rowland, Franklin Mosha, Jovin Kitau

**Affiliations:** 1 Department of Medical Parasitology and Entomology, Kilimanjaro Christian Medical University College (KCMUCo), Moshi, Tanzania; 2 Pan African Malaria Vector Research Consortium (PAMVERC), Moshi, Tanzania; 3 Department of Disease Control, London School of Hygiene and Tropical Medicine (LSHTM), London, WC1E 7HT UK; 4 World Health Organization, Country Office, P.O. Box 9292, Dar es Salaam, Tanzania

**Keywords:** Malaria, Invasive species

## Abstract

To control pyrethroid-resistant malaria vectors, Indoor Residual Spraying (IRS) and Long-Lasting Insecticidal Nets (LLINs) that include additional ingredients to pyrethroid are being developed. Same progress needs to be made to the pyrethroid-treated blankets, which are more compatible with shelter structures found in emergency settings such as displaced populations. In the current study, efficacy of blankets treated with permethrin and piperonyl butoxide (PBO) was evaluated against pyrethroid-resistant *Anopheles gambiae* sensu stricto. Efficacy was compared with that of Olyset LLIN, Olyset Plus LLIN and untreated blanket in terms of mortality and blood-feeding inhibition against pyrethroid-resistant *Anopheles gambiae* mosquitoes. The current study indicates that, in emergency shelters such as migrant and refugee camps where LLINs cannot be used, PBO–permethrin blankets may provide protection against resistant mosquitoes if widely used. No side effects related to the use of the treated blankets were reported from the participants. These results need validation in a large-scale field trial to assess the epidemiological impact of the intervention, durability and acceptability of this new vector control strategy for malaria vector control.

## Introduction

Countries affected by wars or natural disasters are disproportionally affected by malaria^1,2^. In situations where migration occurs from areas of low to high transmission, non-immune refugees and internally displaced populations become highly vulnerable to malaria^3^. Traditionally, a critical determinant of survival for the affected human population in the early stages of a disaster is provision of shelter. This has been restricted to the distribution of untreated blankets, sheets and tents as a means of warmth^4,5^. These provisions are limited in amount and often untreated, therefore cannot provide protection against disease vectors such as mosquitoes. In Tanzania, reports indicate that malaria remains the main cause of morbidity among children under five across all three major refugee camps, Nduta, Nyarugusu and Mtendeli, accounting for about 25 per cent of all morbidity in the camps^6^. The burden of mosquito-borne diseases in refugee camps together with the projected statistics that indicate an increased displacement in Africa^7^, highlight a need to replace untreated blankets with insecticide-treated blankets to control malaria vectors hence adding vector protection benefit in these populations. Several studies have evaluated efficacy of insecticide treated blankets/sheets focusing on pyrethroid treated fabrics among susceptible mosquito populations^8,9^. In Central and South Asian situations, blankets, treated with permethrin have provided personal protection and reduced malaria risk by over 60% among the Afghan refugees^10^.

Pyrethroids remain ideal insecticides for treating nets and other fabrics owing to their low cost, longer residual activity and low mammalian toxicity^11,12^. However, resistance to pyrethroids has been reported in all major malaria vectors in 27 countries across sub-Saharan Africa including Tanzania^13,14^, highlighting the need for alternative insecticides or additional active ingredients in order to improve and sustain the success of malaria control programmes^15,16^. Two primary mechanisms are implicated in conferring resistance to malaria vectors: target site resistance mutations such as knock-down resistance (*kdr*) that occur at the site of insecticide action and metabolic resistance. Metabolic resistance is among physiological changes that occur in mosquito populations based on increased or modified system of cytochrome p450 mono-oxygenases, glutatione-S-transferases and esterases that prevent insecticide from reaching its intended site of action^12^. Metabolic resistance is however more complex than target-site mechanism of insecticide resistance.

Piperonyl butoxide (PBO) is a synergist that enhances the efficacy of pyrethroid insecticides against resistant *Anopheles* mosquitoes by inhibiting enzymes involved in detoxification of those insecticides^17^. Between 2010 and 2020, about 29 countries and 283 sites reported the ability of PBO to fully restore susceptibility of malaria vectors to pyrethroids^18^.

The current availability of factory treatment of fabrics and technology of incorporating PBO in addition to permethrin could result in large-scale production with long-lasting efficacy and protection against pyrethroid resistant vectors at low additional cost^19^. The permethrin–PBO (Skintex**)** blankets manufactured by Pulcra Chemicals LLC Ltd, USA are industrially coated with permethrin (1.25 g/m^2^) and piperonyl butoxide (PBO) at 0.625 g/m^2^. The previous version of these blankets were treated with pyrethroids only and registered by Environmental Protection Agency in the United States. In Tanzania the Tropical Pesticide Research Institute has registered the pyrethroid-treated blankets (IN/0956) for public use. The new blanket version described in this paper is coated with PBO in addition to permethrin, which slowly diffuses to maintain a constant surface concentration. Blankets treated with such a mixture may provide protection against both susceptible and metabolically resistant mosquitoes^20^. This study evaluates the efficacy of a PBO–permethrin blanket against pyrethroid resistant *Anopheles gambiae* sensu stricto (s.s).

## Results

### Regeneration time (RT)

**Figure 1:**
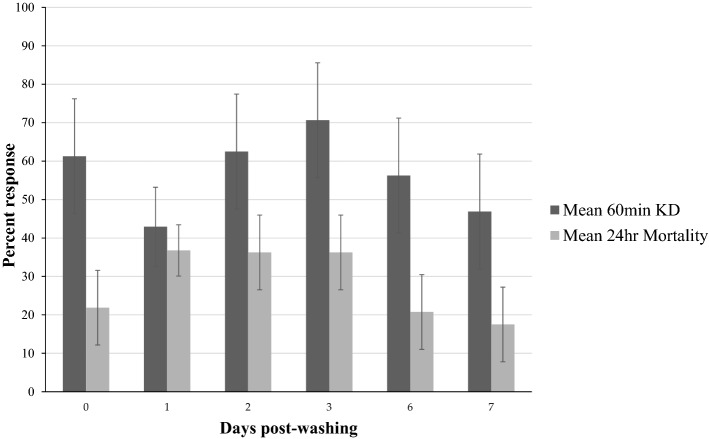
Regeneration time for PBO–permethrin blankets against *An. gambiae* Kisumu. Each bar in the graph represents a mean of ten replicates of 4 blanket pieces cut from 4 individual whole blankets.

At zero wash, mortality and knock-down (KD) in susceptible *An.gambiae* s.s Kisumu were 21.9% and 61.3% respectively. Following three consecutive washes (on the same day) with respective tests afterwards, KD dropped from 61.3% (day 0) to 42.9% (day 1) post washing then increased subsequently to 62.5% and 70.6% day 2 and 3 respectively before declining to56.3% and 46.3% onday 6 and 7 respectively. However, the changes in KD rates were not significantly different since the error bars overlap (Fig. 1). Surprisingly, the percentage mortality did not conform to expected insecticide bioavailability dynamics. Before washing the nets, mortality was very low i.e. 21.9%, increasing to 36.8% on day 1 post washing. The mortality rate remained almost the same (36%) from day 1 to day 3 before declining to 20.8% and 17.5% on day 6 and 7 respectively (Fig. 1). Since KD has a longest RT and follows the expected dynamic of insecticide bioavailability in the treated fabrics, the RT for the PBO–permethrin blanket was confirmed to be 2 days.

### Wash resistance: cylinder assay

Only at 5 washes did the permethrin blanket induce more than 95% KD when tested against the susceptible Kisumu strain. At the 15 washes the blanket induced 88% KD and 18% mortality against the susceptible Kisumu strain. Nevertheless, less than 80% KD was observed for all other washes against both susceptible and a pyrethroid-resistant Muleba-Kis strain^21^, at an exposure time of 3 min in the cylinder bioassay. On the other hand, PBO–permethrin blanket reached 95% KD at both 0 and 5 washes against susceptible Kisumu strain. At 10 times washes, the PBO–permethrin blanket reached 77% KD, higher than permethrin-only blanket by 30%. At t 15 washes the PBO–permethrin blanket induced only 12% KD compared to 88% KD induced by the permethrin-only blanket. However, the PBO–permethrin induced 16% mortality against the susceptible Kisumu strain equivalent to permethrin-only blanket. Both permethrin-only and PBO -permethrin blanket did not reach the 20% KD when the pyrethroid resistant mosquitoes were used. Each bar of a graph represents a mean KD of 4 replicates. Also, mortality of resistant Muleba-Kis mosquitoes induced by both blankets was less than 25%.

**Figure 2:**
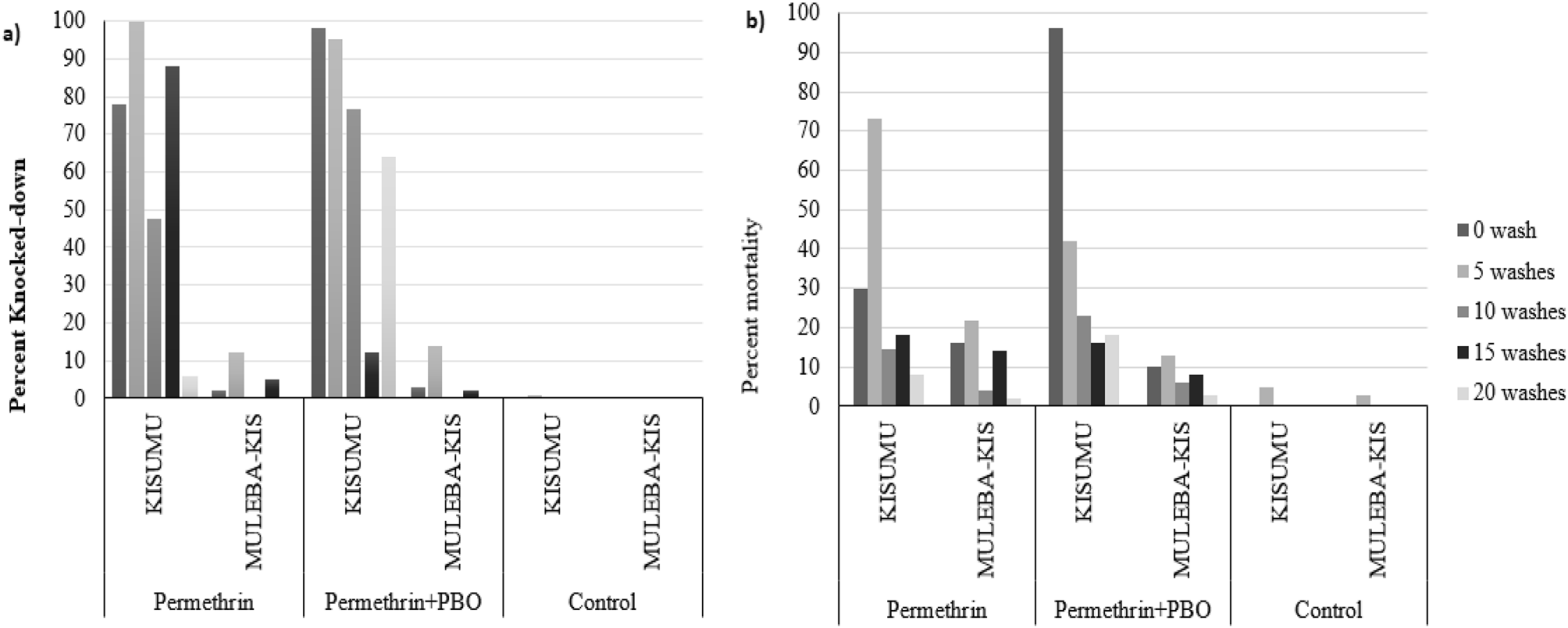
Efficacy of PBO–permethrin blanket in terms of (**a**) KD and (**b**) mortality against susceptible Kisumu and resistant Muleba-Kis strains.

In terms of mortality, only PBO–permethrin blanket unwashed reached the 80% against susceptible Kisumu, while all washes were below the cut-off for both permethrins-only and PBO–permethrin treated blankets. The efficacy of PBO–permethrin in terms of KD and mortality effect after 0, 5, 10, 15 and 20 washes are shown in Fig. 2a,b.

**Figure 3:**
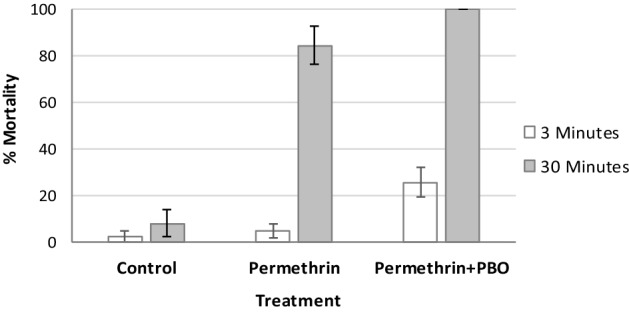
Mortality rates for susceptible strain for 3 min exposure versus 30 min exposure from different blanket treatments. Error bars are equivalent to 95% confidence intervals.

Due to low mortality and avoidance to contact the treated material, cylinder assays were repeated with 30 min exposure for pieces washed 10 times against susceptible *An. gambiae* Kisumu. Both PBO–permethrin and permethrin-only blankets gave mortality above 80% which is above the WHO cut-off point Fig. 3.

### Wash resistance: tunnel test assay

**Figure 4:**
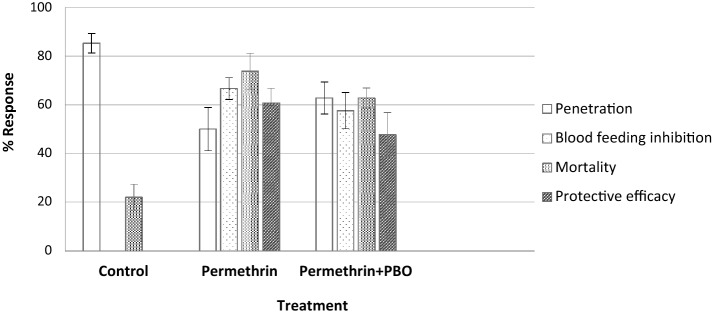
Penetration, blood-feeding, mortality and protective efficacy for different blanket treatments in tunnel tests against *An. gambiae* Kisumu. Error bars are equivalent to 95% confidence intervals.

The PBO–permethrin pieces that were washed 10 times with mortality closest to the mean mortality in the cylinder assay were used for tunnel test against susceptible *An. gambiae* Kisumu. For the control tunnel, blood feeding was 80% which is above WHO cut off point (≥ 50%).The penetration rate was above 85% in the control tunnel. On the other hand, both permethrin and PBO–permethrin blanket showed reduced blood feeding by over 50% relative to the control. The PBO–permethrin reduced mosquito penetration by 27% relative to the negative control. The protective efficacy (protection resulting from reduced mosquito penetration, blood-feeding inhibition and mortality effects) by permethrin and PBO–permethrin blankets was higher than the control arm (Fig. 4).

**Figure 5:**
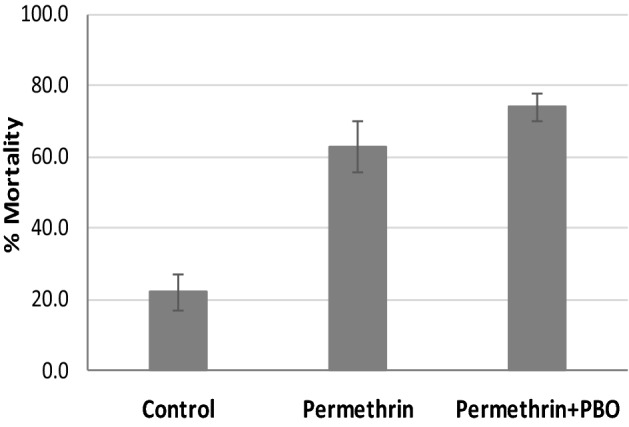
Mortality rates of resistant *Anopheles gambiae Muleba*-Kis after exposure to different blanket treatments in tunnel tests. Error bars are equivalent to 95% confidence intervals.

The PBO–permethrin blanket in the tunnel test induced mortality above 70% against pyrethroid resistant *An. gambiae* Muleba-Kis, which is 10% higher than the permethrin only blanket (Fig. 5).

### Experimental hut trial

#### Release-recapture

All treatments killed (immediate mortality) significantly more mosquitoes (31.7%–87.3%) than the untreated blanket (*p* < 0.05). The higher mortality was observed with the Olyset Plus (94.0%) followed by combination of PBO–permethrin blanket with Olyset net (77.9%), unwashed PBO–permethrin blanket (53.7%), Olyset net (56.3%) and lastly by the washed PBO–permethrin blanket (38.4%). Unlike the washed treated blankets which induced less mortality than the standard Olyset net, the unwashed treated blankets induced similar mortality as the standard pyrethroid Olyset net (Table 1).

**Table 1:** The overall percentage mortality of *Anopheles gambiae* Muleba-Kisumu in the experimental huts.

Treatment type	Washes	N	Total dead	% dead	AOR	95% CI	*p*-value	z-value
Positive control (Olyset LLIN)	20	646	364^a^	56.3%	1			
PBO–permethrin blanket and Olyset LLIN	10,20	648	505^b^	77.9%	3.67	(1.58–8.53)	0.003*	3.02
Olyset Plus LLIN	20	684	643^c^	94.0%	19.69	(7.80–49.68)	< 0.001*	6.31
Untreated blanket	10	641	43^d^	6.7%	− 0.04	(0.02–0.09)	< 0.001*	− 7.29
PBO–permethrin blanket	0	637	342^a^	53.7%	0.42	(0.38–2.02)	0.767	− 2.06
PBO–permethrin blanket	10	627	241^e^	38.4%	0.88	(0.18–0.95)	0.039*	− 0.30

N is total mosquito released; AOR is adjusted odds ratio for random effects; CI is confidence interval.* Statistically significant. Values along a row bearing the same letter label are not significantly different (*p* > 0.05).

**Figure 6:**
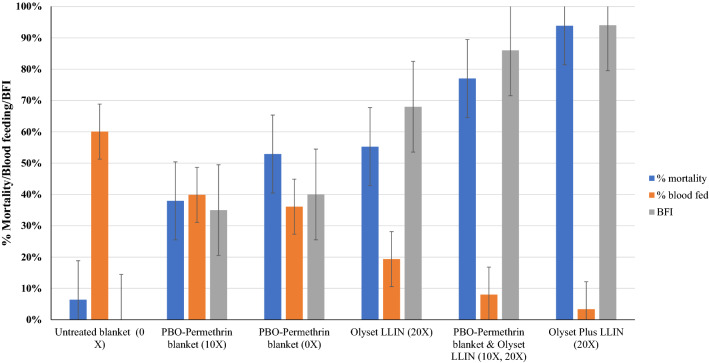
Mortality and blood feeding rates for different treatment arms. Error bars are equivalent to 95% confidence intervals.

As for mortality, blood feeding inhibition was the highest in the treatment with PBO–Pyrethroid LLIN and the blanket + net (Fig. 6). High and significant blood feeding inhibition (BFI) rates were detected with all treatments compared to the negative control (*p* < 0.05). Olyset Plus washed 20 times induced the highest BFI rates (94.0%) followed by washed PBO–permethrin blanket combined with washed Olyset Net (86.5%), Olyset Net (67.5%), unwashed PBO–permethrin blanket (40.3%), washed PBO–permethrin blanket (35.1%) and Table 2.

**Table 2:** The percentage blood feeding of *Anopheles gambiae* Muleba-Kisumu in the experimental huts.

Treatment type	Washes	N	N blood fed	% fed	AOR	95% CI	*p*-value	z-value
Positive control (Olyset LLIN)	20	646	125^a^	19.3%	1			
Treated blanket & Olyset LLIN	10,20	648	52^b^	8.0%	0.35	(0.11–1.11)	0.075	− 1.78
Olyset Plus LLIN	20	684	23^c^	3.4%	0.13	(0.04–0.43)	0.001*	− 3.31
Untreated blanket	10	641	385^d^	60.1%	9.49	(3.11–29.00)	< 0.001*	3.95
Treated blanket	0	637	230^e^	36.1%	2.39	(0.78–7.36)	0.129	1.52
Treated blanket	10	627	250^e^	39.9%	3.43	(1.12–10.50)	0.031*	2.16

N is total mosquito released; AOR is adjusted odds ratio for random effects; CI is confidence interval; * Statistically significant. Values along a row bearing the same letter label are not significantly different (*p* > 0.05).

#### Adverse effects reporting

In this study none of the sleepers reported any apparent side effect due to skin contact with treated blankets.

## Discussion

The present study, explored the efficacy of PBO–permethrin blanket against pyrethroid-resistant *An. gambiae.* Treated blankets by themselves were more protective than untreated blanket in killing mosquitoes and preventing against mosquito bites.. Similar level of protection was obtained with treated and unwashed PBO–permethrin blanket compared to standard Olyset LLIN, but this decreased with increasing number of washing. The insecticide wash fastness from the blanket fabrics was also reported in a separate study^8^. However, the findings of this study were limited by the fact that chemical analysis was not done to determine the chemical content in the blankets before and after washing. Chemical analysis would enable determination of retention, bioavailability and replenishment of both permethrin and PBO in the treated blankets.

The best effect was observed when the PBO–Permethrin treated blanket was associated with a standard LLIN, where the performance in preventing bites and killing mosquitoes exceeded that of the standard net, and only less to the effect induced by the Olyset Plus in terms of mortality and slightly as well in feeding inhibition. This could partly be attributed to PBO treatment on blankets complimenting effect of permethrin treatment and a physical barrier provided by the standard pyrethroid net. However, treated blankets on their own, only offer a moderate protective effect against the *Anopheles gambiae* in the experimental hut trial. This could be attributed to high resistance to permethrin due to target-site modifications and possible elevated metabolic mechanisms in the mosquitoes^21^. Significantly higher mortality rates is anticipated for PBO–permethrin blankets in areas with low pyrethroid resistance as has been observed elsewhere for Olyset nets^22,23^. The observation that, its effect was moderate against pyrethroid resistant *An. gambiae* mosquitoes in experimental huts is likely to be a consequence of low surface concentration of both permethrin (insecticide) and PBO (synergist) and mode of treatment. Further studies are recommended for optimizing the effective surface concentration of insecticide and synergist under simulated conditions. In this study, permethrin-only treated blankets were not included as one of the treatments in the experimental huts trial to show the advantage of PBO blanket against pyrethroid resistant mosquitoes. Nevertheless PBO–permethrin blanket performed better than permethrin-only blanket in laboratory wash resistance experiments.

The present study suggests an advantage for combining PBO–permethrin blanket and pyrethroid LLIN in terms of blood-feeding inhibition (27.7%) and mortality (19%) compared to pyrethroid standard LLIN. This could justify promoting this strategy in the future in areas where malaria vectors are resistant to pyrethroids. Development of a blanket incorporating a pyrethroid with a synergist is promising against resistant malaria vectors, especially in settings where LLINs are less practical^19,24^ or in situations where the available nets are pyrethroid only and there is a considerable risk of malaria transmission due to reduced sensitivity to pyrethroid-resistant vectors. The present study has also shown reduced efficacy of pyrethroid treated nets compared to the PBO–Pyrtehoid LLIN against pyrethroid-resistant malaria vectors, which confirms previous findings in several African countries^25,26^.

Athough blankets can not be compared to nets in all aspects due to their basic differences and mode of application, still both, when treated can be used to kill and deter mosquitoes. The intrinsic difference exists between treated blankets compared to the LLINs, such as lack of complete physical barrier against biting mosquitoes, and requirement to provide full protection only when a sleeper is covered. Sleeper might not always be covered by the blanket, even at cool temperature^8^, partially attributing to slightly low blood feeding inhibition induced by treated blanket compared to standard net. On the other hand, the use of blanket might obscure the diffusion of human odor and other cues that go through easily with a net and mesh, and hence in an experimental hut using free flying mosquitoes, a lower number is expected to be attracted in blanket arms compared to net arm^8^. Nevertheless, the PBO–permethrin blankets can be improved and deployed to control malaria vectors. Following the deployment of PBO–permethrin blankets, it is expected that refugees and displaced populations, as well as complex emergences, will be protected from malaria..

In this study no apparent side effect due to skin contact with treated blankets was reported. The blankets are that were tested are treated with either permethrin (pyrethoid) only or both permethrin and PBO. While pyrethroids have been shown to have very low mammalian toxicicity, but are toxic to insects and knock them down (kill them) even at very low doses^11^, PBO is practically non-toxic to birds and mammals. According to WHO^27^, a PBO-treated ITN with a concentration of 1000 mg/m^2^ (fabric weight 40 g/m^2^ and 25 g/kg net) and a Wash Resistance Index of 90% can be used safely for its intended use as a vector control product.

In the laboratory regeneration experiments, the KD reached the initial efficacy on the second day and the mortality reached a plateau above the initial level after one day. There was however, no anticipated reduction and then increase in mortality before and after washing. This could probably be attributed to incosistent insecticide migration to the surface. This was not observed in the KD pattern. On the other hand in the wash resistance experiments, PBO–permethrin blanket did not induce a significant mortality or KD when washed more than 5 times at 3 min exposure assay against either susceptible or resistant *An. gambiae*. Since the susceptibility of the Kisumu strain is quartery characterized and confirmed, the observed low mortality could be attributed to low surface concentration bio-available in a brief exposure period, repellence nature of the incorporated permethrin and probable avoidance behaviour^28,29^. Increasing the exposure time in the cylinder assays from 3 to 30 min resulted to higher KD and mortality for both susceptible and resistant mosquitoes. This could be a result of increased contact number and duration to treated surface even at a low surface insecticide concentration. The maximum number of washes for blankets was restricted to 10, as higher washes did not induce substantial mortality and KD. In typical refugee or emergence camps, which are characterized with low per capita availability of water^30,31^ it is more likely for blankets to be less frequently washed compared to other settings where water is available. Furthermore, in tunnel tests, blankets treated with either permethrin alone or permethrin with PBO provided significant protective efficacy in terms of blood-feeding inhibition, and reducing penetration of mosquitoes compared to untreated blankets.

The combination of PBO–permethrin blankets with Olyset induced significantly higher mortality, exophily and blood-feeding inhibition against the resistant mosquitoes than either the Olyset Net or PBO–permethrin blankets alone. This finding is similar to the standard permethrin blanket previously recommended for deployment only in areas with pyrethroid susceptible vectors^14^.

This study also demonstrates the potential benefit of incorporating an insecticide active ingredient and a synergist into a blanket to better control malaria vectors and/or complementing the Olyset nets in controlling the resistant mosquitoes. Nevertheless, results in this study highlight a need to further optimize the technology to incorporate active ingredient in the blankets to ensure the insecticide is retained in the material even after washes. This would win a race towards increasing vector control coverage and control transmission in settings where current primary tools are impractical. However, while the results calls for further technological adjustment to develop this tool, there is need for validation by a large-scale field trial to assess the epidemiological impact of the intervention, durability and acceptability of this new vector control strategy for malaria vector control.

## Methods

All methods were performed in accordance with the relevant guidelines and regulations.

### Study site

The laboratory experiments on regeneration and wash resistance were conducted at the KCMUCo-PAMVERC Insecticide Testing Facility; while experimental hut study was carried out at Harusini, the facility’s field site located at Mabogini village (S03˚22.764’ E03˚720.793’), adjacent to Lower Moshi rice irrigation scheme in north-eastern Tanzania. The dominant vector at this site is *An. arabiensis* with moderate level of resistance to pyrethroids conferred by both oxidase and esterase activities^32^. In this study, pyrethroid-resistant laboratory reared *An. gambiae* Muleba-Kis mosquitoes were released into the huts for the release-recapture experiment.

#### Test systems

Non-blood fed, 2–5 day old females of susceptible *An. gambiae* s.s. Kisumu strain and pyrethroid resistant *An. gambiae* s.s Muleba-Kis strain were used for the evaluation of efficacy in the laboratory (phase I). The Muleba-Kis strain has been colonized for more than 8 years and it is resistant to permethrin with fixed L1014S *kdr* frequency and metabolic resistance through increased oxidase activity has also been reported^21^. Only *An. gambiae* s.s Muleba-Kis were used in release-recapture experiments. The Kisumu strain is fully susceptible to insecticides and free of any detectable insecticide resistance mechanisms. The strain originated from Kisumu, Kenya and has been colonized for many years in laboratory. At the KCMUCo-PAMVERC Moshi insectary, the adult Kisumu strain mosquitoes are reared at a temperature of 24–27 °C, 75 ± 10% relative humidity (RH) and maintained under a dark:light regime of 12:12 h. The Muleba-Kis mosquitoes used for the release-recapture experiments were reared in the field insectary under ambient temperature and relative humidity and treated as previously explained^21^. The susceptibility status of these colonies is checked every three months using WHO susceptibility test^33^ and, CDC bottle bioassay test^34^. The colonies are regularly genotyped for *kdr* mutations using TaqMan assays^35^. To maintain the resistance of Muleba-Kis, larvae are frequently selected with alpha-cypermethrin.

#### Regeneration time

To determine the regeneration time of the insecticide-treated blankets, blankets were cut into 25 × 25 cm pieces and tested before washing and then washed and dried three times consecutively following WHO recommended procedures for LLINs^36^. The pieces were then re-tested after one, two, three, six and seven days post-washing using WHO cylinders against susceptible *An. gambiae* s.s (Kisumu).

Graphs for 24-h mortality and 60 min knock down (KD) correlating to insecticide bioavailability, as measured by 3 min exposure in cylinder bioassays, were established before and after washing blanket pieces three times consecutively in a day, and tested within a maximum of seven days post-washing. The time in days required to reach initial mortality or 60 min KD plateau is the period required for full regeneration of insecticide-treated blanket.

#### Wash resistance

WHO cylinder bioassays^36^ were used to assess the wash resistance for the blanket pieces washed 0, 5, 10, 15 and 20 times at the intervals equivalent to the regeneration time. Four pieces cut from 4 permethrin and 4 untreated blankets were used as positive and negative control respectively, against 4 pieces cut from 4 PBO–permethrin blankets.

#### Bioassay procedures

Five, non-blood fed, 2–5 day old *An. gambiae* Kisumu or *An. gambiae* Muleba-Kis mosquitoes were exposed for 3 min or 30 min to blanket pieces in WHO cylinder. Bioassays were carried out at 27 ± 2 °C and 75 ± 10% RH. Knock-down was scored after 60 min post-exposure and mortality after 24 h. Fifty mosquitoes (5 mosquitoes per cylinder) were used on each 25 × 25 cm piece of blanket sample. After exposure, the mosquitoes were held for 24 h with access to 10% glucose solution in the paper cups covered with a net material. Mosquitoes exposed to untreated blanket were referred as a negative control.

#### WHO tunnel test method

Blanket pieces which recorded ≤ 80% mortality in cylinder bioassay were tested in the tunnel assay using WHO guidelines. The tunnel was made of an acrylic square cylinder (25 cm in height, 25 cm in width, and 60 cm in length) divided into two sections using a blanket-covered frame fitted into a slot across the tunnel. During the assays a guinea pig was held in a small wooden cage (as a bait) in one of the sections and 50, non-blood fed, female *An. gambiae* Kisumu or *An. gambiae* Muleba-Kis aged 5–8 days were released in the other section at dusk and left overnight (13 h) for experimentation at 27 ± 2 °C and 75 ± 10% RH. The blanket surface was deliberately holed (nine 1-cm holes) to allow mosquitoes to contact the blanket material and penetrate to the baited chamber. Treated blankets were tested concurrently together with an untreated blanket. Scoring for the numbers of mosquitoes found alive or dead, fed or unfed, in each section were done in the morning. Mosquitoes found alive were removed and held in paper cups with labels corresponding to each tunnel sections under controlled conditions (25–27 °C and 75–85% RH) and fed on 10% glucose solution to monitor for delayed mortality post exposurely. Outcomes recorded were: mosquito penetration, blood feeding and mortality.

#### Washing of blankets and whole nets for hut trial

Blankets and whole nets were separately washed following WHOPES guidelines. In brief, each blanket/net was washed in Savon de Marseilles soap solution (2 g/L) for 10 min: 3 min stirring, 4 min soaking, then another 3 min stirring. This was followed by 2 rinse cycles of the same duration with water only. The water pH was 6 for all washes. The mean water hardness was within the WHOPES limit of ≤ 89 ppm. All nets used in the experimental hut study were cut with holes (4 cm × 4 cm) to simulate the conditions of a torn net. While nets were washed 20 times as per guidelines, blankets were only washed 10 times. To simulate a situation in emergence situations where washing is less frequent due to water scarcity^30,31^.

#### Experimental hut trial:experimental hut design

Experimental hut study was done in Lower Moshi using typical East African experimental huts design as described in the WHOPES^35^. Huts were constructed with brick walls and featured with cement plaster on the inside and a ceiling board, a metal iron sheet roof, open eaves with window and veranda traps on each side and window traps. Slight modifications from the original structure were made by installing metal eave baffles on two sides. The baffles allow mosquito entry but prevent exits. The window traps were used to collect mosquitoes that tend to exit the huts.

#### Test item labelling, washing and perforating

Both blankets and LLINs for the trial were distinctively labelled with fabric labels that withstand washes. For wash resistance, the blankets and nets were separately washed according to a protocol adapted from the standard WHO washing procedure^36^ at the interval equivalent to the regeneration time established in the laboratory for blanket and LLIN respectively. Before testing in the experimental huts, all nets were deliberately holed i.e. 30 holes measuring 4 × 4 cm were made in each net, 9 holes in each of the long side panels, and 6 holes at each short side (head- and foot-side panels) to enhance blood-feeding on the control arm.

#### Test items packaging

Each blanket and net were sealed in a plastic bag and then packed in the large plastic container. Each container was labelled for a single treatment to avoid cross contamination between test items.

#### Experimental hut decontamination

A cone assay with 10 susceptible mosquitoes was performed on one wall per hut to rule out any contamination of the wall surface. Only huts with 24 h mortality of susceptible mosquitoes < 10% were used.

#### Predator check

Experimental hut rooms, verandas and exit traps were searched for ants. Where found, clean petri dishes containing boric acid-sugar mixture (1:1) was used to put down ants. Subsequently, the ant numbers were monitored by using dead mosquitoes as bait. The trials started only when the mosquito baits were still in the huts for 24 h. Rooms and verandas of all huts were checked for spiders and spider webs and the baffles were checked for the presence of lizards.

#### Leakage check

All veranda walls and screens were checked for cracks and holes respectively and repaired as appropriate. The baffles in the ‘entry eave gap’ sides and the eave gap in the ‘exit eave gap’ sides were checked for uniformity targeting the gap level of 5 ± 2 cm when measured from the inside of the hut. Water moats around the huts were checked for leakage and repaired when found leaky. Exit traps were checked for holes and repaired, cleaned and hung back up. Any gap around the window trap was sealed with clean cotton wool.

#### Volunteer recruitment, medical care and assessment of adverse events

Six adult male volunteer sleepers were consented, recruited and trained for the study; the volunteers slept individually in the huts every test night from 19:00 to 06:30 h. Before the study, all sleepers underwent a medical checkup for malaria and symptoms for COVID-19. Every Monday and Friday morning before mosquito collection, sleepers were asked if they experience any apparent side effect due to skin contact with treated blankets.

#### Release-recapture experiment

The efficacy of treated blanket was evaluated against pyrethroid resistant *An. gambiae* Muleba-Kis which were released in each experimental hut early in the evening and re-captured the next morning. During the 36 nights of collections, a total of 4320 *An. gambiae* s.l. Muleba-Kis were used for the release-recapture study with a recapture success rate of approximatively 90%. Six trial arms: untreated blankets (washed 10 times), Olyset LLIN (washed 20 times), Treated Blanket & Olyset LLIN roof-treated IG2 (10 times and 20 times washed respectively), Olyset Plus LLIN (washed 20 times), treated blanket (10 times washed) and unwashed treated blanket were tested in separate huts for 36 collection nights from July to August 2021.

#### Rotation of treatments and sleepers

A full 6 × 6 Latin square rotation schedule was automatically generated^37^. Treatments were rotated between experimental huts every six days, and sleepers every day to limit any location bias resulting from differences between sleepers, huts and treatments. Three replicates per treatment arm were used and exchanged daily to capture any variation within treatments. The huts were cleaned and left for ventilation every 7th day before the next rotation.

In each hut one volunteer slept every trial night from 19:00 h to 06:30 h the following morning and were instructed to sleep under the net (Olyset or Olyset Plus or untreated) and/or to cover themselves with a blanket (untreated or treated). During each trial night supervisors performed two inspections and scored the level of blanket coverage on sleepers, following the scoring chart adopted from Kitau et al.^8^.

#### Mosquito release and collection procedures

Blankets and LLINs were evaluated in the Mabogini experimental huts from June to July 2021, corresponding to 36 nights of collections from 6 huts. In each hut, 20 blood-unfed, 2–5 days old female *An. gambiae* Muleba-Kis were released inside the hut and collected in the morning. Collection of the mosquitoes started at the negative control hut and proceeded to the single insecticide treatment arms, then to the permethrin–PBO positive control, then lastly PBO–permethrin blanket. The mosquitoes were first collected from the floor, inside the nets, walls, ceiling and exit traps. The time spent on collecting mosquitoes was strictly followed with an acceptable variation of time to prevent collector bias between huts; exit traps (3 min), room floor (5 ± 1 min), room walls and ceiling (5 ± 1 min) and in the net (6 ± 1 min).

#### Mosquito scoring

Two technicians were involved in scoring, mosquitoes identification and filling the data forms. Mosquitoes were systematically scored by species (*Anopheles* then *Culex*), by location (floor, net, wall, ceiling, window trap), gonotrophic and alive/dead status. Alive mosquitoes were placed in small paper cups and provided access to 10% glucose solution. Temperature and RH were also recorded during scoring. Scoring was repeated after 24 h for delayed mortality.

#### Outcome measures

Each net treatment, blanket treatment or blanket in combination with net was evaluated for blood feeding inhibition and induced mortality as primary outcomes. Repellence was also scored as a secondary outcome. Immediate and delayed mortality were determined as the proportions of total mosquitoes released into the hut and collected dead in the morning (immediate mortality) or when caught alive and die at 24 h. Blood-feeding inhibition was determined as reduction in blood-feeding in treatments relative to the negative control hut. Repellence (induced exophily) was determined as a proportion of mosquitoes collected from the exit traps from treated huts relative to proportion caught in exit traps of negative control hut.

#### Statistical methods

Statistical analyses were performed using Stata 16 (Stata Corp LP, College Station, TX, USA). Mortality from the cylinder bioassay and tunnel tests were expressed as percentages and 95% confidence intervals was calculated. For the experimental hut trial, data were entered in an excel database and transferred to Stata for processing and analysis. In brief, proportion of blood feeding inhibition, exophily and overall killing effect were calculated. Data were analysed with a linear mixed model that included the adjacent hut treatment as independent variable and negative binomial regression for count data. The night of capture, the hut and the sleeper were considered as fixed effects. The primary criteria in the evaluation were blood-feeding inhibition and mortality, with exophily as a secondary outcome.

Percentage mortality was estimated using the formula

$$\left[ {\left( {{\text{N}}_{{\text{d}}} } \right){\text{/N}}_{{\text{t}}} } \right]\, \times \,100\%$$

where *N*_d_ = the number of mosquitoes found dead in hut, *N*_t_ = total number of mosquitoes released in hut.

Percentage blood feeding inhibition (BFI) was estimated using the formula:

$$\left[ {\left( {F_{{\text{c}}} - F_{{\text{t}}} } \right)/F_{{\text{c}}} } \right] \times 100\%$$

where *F*_c_ = number of mosquitoes found fed in untreated control hut, *F*_t_ = number of mosquitoes found fed in treated hut.

Pertentage induced exophily was estimated using the formula

$$\left( {{\text{Nv}}/{\text{Nt}}} \right) \times {1}00$$

where Nv = number of mosquitoes found in verandah, Nt = the total number of mosquitoes found inside the hut and verandah.

The overall protective efficacy (%) of the treatment considered that a significant number of mosquitoes were inhibited from penetration and not killed by the treatment and was estimated using the formula

$$\left[ {\left( {D_{t} {-}D_{c} } \right)/P_{c} } \right] \times 100$$

where *D*_*t*_ is the total number of mosquitoes found dead in the treatment tunnel, *D*_*c*_ is the total number of mosquitoes found dead in the control tunnel, *P*_*c*_ is the total number of mosquitoes penetrating in the control tunnel.

### Institutional review board statement

The required data were collected after obtaining ethical clearance from Kilimanjaro Christian Medical University College (Research Ethical Clearance Certificate No. 2512), and it is part of the large ongoing research program that was reviewed and approved by the Tanzania National Institute for Medical Research (NIMR/HQ/R.8c/Vol.1/554). Written informed consent in local language (Swahili) was obtained from the participants before participation.

### Informed consent

Informed consent was obtained from all subjects involved in the study.

## Data Availability

The datasets supporting the conclusions of this article are included within the article (and its additional file(s)).
